# Validation of the Organizational Dehumanization Scale in Spanish-Speaking Contexts

**DOI:** 10.3390/ijerph19084805

**Published:** 2022-04-15

**Authors:** Eva Ariño-Mateo, Raúl Ramírez-Vielma, Matías Arriagada-Venegas, Gabriela Nazar-Carter, David Pérez-Jorge

**Affiliations:** 1 Department of Psychology, European University of Valencia, 46010 Valencia, Spain; eva.arino@universidadeuropea.es; 2 Department of Psychology, Universidad de Concepción, Concepción 4030000, Chile; rauramir@udec.cl (R.R.-V.); matarriagada@udec.cl (M.A.-V.); gnazar@udec.cl (G.N.-C.); 3 Department of Didactics and Educational Research, University of La Laguna, 38200 San Cristóbal de La Laguna, Spain

**Keywords:** organizational dehumanization, scales, Spanish, validation

## Abstract

The objective of this study is to validate Caesens, Stinglhamber, and Demoulin’s (2017) organizational dehumanization scale (ODS) in a Spanish-speaking sample. A sample of 422 employees (49.3% women and 50.7% men) from Chile answered an online questionnaire comprised of measures of organizational dehumanization and job satisfaction, organizational citizenship behaviors, and authentic leadership. To analyze the structure of the ODS, the sample was divided into two random subsamples and exploratory and confirmatory factor analyses were carried out. In addition, reliability and criterion validity were tested. As a result, the scale was composed of one factor. One item was eliminated due to its factor loading. The internal consistency was good (α = 0.92; ω = 0.92). The correlations between ODS, job satisfaction, organizational citizenship behaviors, and authentic leadership were statistically significant, from medium to high magnitude, indicating a reasonable degree of criterion validity. In conclusion, the Spanish version of the ODS shows adequate psychometric properties and can be useful for making progress on the understanding of organizational dehumanization and evaluating the organizational dehumanization in Spanish-speaking context.

## 1. Introduction

Organizational dehumanization refers to “the experience that workers have when they feel like an object within their organization” [1] and responds to the need to better understand the underpinnings behind the view of workers as instruments or work tools, that is, as a “mean” for organizational objectives instead of an “end” [2]. The construct was born of the sociological work of Marx [3], Durkheim [4], and Weber [5] and in the area of social psychology, with the theory of infrahumanization [6,7] and the dual model of dehumanization [8]. Both theories analyze explanations of humanity in ingroups and outgroups, asserting that people attribute a human essence to themselves and their own groups and deny these human characteristics to other groups [6,7,8,9]. On the one hand, studies on infrahumanization base their main hypothesis on the idea that people have a stronger association between secondary emotions toward their ingroup and restrict the possibility of experiencing these human emotions to the outgroup. This difference does not happen with the primary emotions that both humans and other animal species have [10,11,12]. Likewise, the dual model of dehumanization developed by Haslam [8] states that the central aspects of the human condition rely on human traits and traits of human nature [13]. Therefore, when a group is characterized in terms of nonhuman traits (e.g., irrational or uncivil), that would indicate that the group is dehumanized in animal form. Features of human nature (HN) differentiate the human being from a machine, and when a group is assigned a lack of emotions or coldness, it would indicate that the group is suffering mechanistic dehumanization [9].

Dehumanization seems to be an increasingly frequent experience in organizations [14]. There are several studies that affirm that mechanization is present in the context of high industrialization in which workers are considered pieces of a gear [8], in the business world, where executives are compared with machines [15], or in the health sector when patients are dehumanized by considering them as machines whose parts need to be repaired [16]. It is increasingly common for organizations and their leaders to perceive workers as robots or tools of their property created for their own benefits and purposes [14], particularly in an era where Industry 4.0 has introduced digital technologies in factories with the aim of transforming production processes and making them more efficient. Dehumanizing implies stripping another person or group of those characteristics that are part of the human essence [6,8]. When a leader has no respect for their workers or perceives them without decision-making capacity or without the possibility of experiencing emotions, they are dehumanized and thus can be treated with indifference or a lack of empathy [17,18]. Dehumanization by the leader causes the worker to be perceived as a totally interchangeable object or tool [19,20].

There are several studies that show the relationship between dehumanization and negative behaviors. In addition, Galinski et al. [21] showed that people who can exert power, such as managers, do not adopt others’ points of view. Power favors interpersonal distance from others and increases deindividualization mechanisms, which are linked to dehumanization [22,23,24]. Rudman et al. [25] affirm that dehumanization increases the use of harmful behavior and justifies the use of violent and harmful methods. It has also been shown that the dehumanization of the worker increases avoidance behaviors and social rejection from management [26] and reduces the intention to provide help [27]. 

Dehumanization causes significant problems for those who suffer it [28]. There are several studies that affirm that dehumanization promotes anxiety or depression and reduces the need for competition and interaction, damaging their well-being [14,29]. Along these lines, studies affirm that dehumanization promotes burnout since workers feel being perceived as an instrument by their supervisor [30,31]. In addition, Christoff [14] states that dehumanization negatively affects the worker’s citizenship behaviors toward colleagues and the organization and leads to emotions of sadness, anger, guilt, and shame. 

It has also been shown that dehumanization affects the ethical climate of the organization. Väyrynen et al. [18] state that employees perceive that they cannot trust their organization when they do not feel they are being treated with respect. In addition, the ethical organizational climate affects performance, quality, trust, and commitment [32,33]. Moreover, Mosso et al. [34] demonstrated how the status of the outgroup influenced dehumanization and how this status is related to system justification beliefs [35].

However, dehumanization in the organizational context can also have positive effects and can be useful in specific contexts. As Christoff [14] stated, since dehumanization increases personal distance and coldness in personal interactions, it can favor the decision-making process and help make better decisions. Another silver lining of organizational dehumanization is described by Lee et al. [36], who found that the distance created by a videoconference, compared to the face-to-face position, facilitated the decisions that entailed a greater risk toward the interlocutors.

Moreover, dehumanization seems to be beneficial in the health sector. Human suffering is a negative stimulus that health workers must go through every day, and for them to humanize their patients would imply a high emotional cost. Organizational dehumanization can reduce these costs and release cognitive and emotional resources, thus, treating the patient as a number or a machine and helping health workers face the demands of being emotionally involved with patients’ suffering [37]. Vaes et al. [38] found that the humanization of the patient positively predicted burnout symptoms, especially for those who had higher levels of direct contact with patients. Along these lines, Haque et al. [16] stated that it is useful to maintain psychological distance when a patient needs a treatment in which pain is infringed.

In response to the need to have a valid measure of this construct in organizational contexts, Caesens et al. [39] developed the scale of organizational dehumanization. This 11-item scale evaluates organizational dehumanization using a Likert-type scale of seven points from 1 (“strongly disagree”) to 7 (“strongly agree”). The present study adapts and validates this scale to the Spanish language to have a valid and reliable measure of this construct in this context. The consequences of dehumanization have been detailed previously and through this scale, it is intended to eliminate linguistic barriers in the study of dehumanization, facilitate the progress in the knowledge of theoretical bases of dehumanization, and perform comparative studies analyzing organizational dehumanization between Spanish- and English-speaking countries. 

There are at least three reasons for the importance and interest of the adaptation and validation of the scale of organizational dehumanization in Spanish. First, it constitutes a new and useful instrument for measuring the mechanistic organizational dimension of dehumanization in work contexts and operationalizes the construct in a simple and clear way. Second, this instrument is the only one available to date, and it incorporates the social and organizational psychology of dehumanization perspectives, which implies a broader and more inclusive view of the phenomenon. Third, the idiomatic adaptation of this instrument fills the research gap regarding the availability of valid and reliable measures of the phenomenon of organizational dehumanization in Spanish-speaking contexts and adapting it makes it available for its potential application in different Spanish-speaking countries. 

The International Test Commission Guidelines for Translating and Adapting Tests were followed and the process of translation, synthesis, reverse translation, expert committee review, and pre-test were achieved. Following these different stages implied a cultural adaptation in the Spanish context. Consequently, this work shows such a process of adaptation to this language, focusing on obtaining empirical evidence of reliability and validity on a sample of 400 workers from different organizations in Chile, representing a wide variety of positions and occupational groups. Finally, this article presents a tool for evaluating dehumanization in organizations in a Spanish-language context that allows leaders to evaluate the perception of workers in how they are treated by the organization. The main focus of this article is to present the initial findings related to the psychometric properties of the scale.

## 2. Materials and Methods

The main objective of the research was to adapt and validate the Organizational Dehumanization Scale from Caesens et al. [39] to the Spanish language to have a valid and reliable measure of this construct in the Spanish-speaking context. Consequently, this work shows such an adaptation process to this language, and the psychometric properties of the instrument were studied, including the reliability and validity in terms of its factor structure and concurrent validity. 

### 2.1. Participants

The sample was obtained in a non-probabilistic way and was composed of 422 Chilean workers from different economic sectors, geographic areas, organizations, and types of work. In total, 1800 surveys were sent out, and with a confidence level of 98%, the test was completed for 422 workers. As an exclusion criterion was stated that the worker has a minimum tenure of six months in the organization so that they could have experienced the environment of their organization. The mean age of the participants was 38.96 years (*SD* = 11.40). Of the total sample, 49.3% were women (*n* = 208) and 50.7% men (*n* = 214). In educational terms, 9.7% (*n* = 41) of the participants had a postgraduate degree, 41.2% (*n* = 174) had a university degree, and 30.1% (*n* = 127) technical degree, while 18.2% (*n* = 77) and 0.7% (*n* = 3) had secondary and postgraduate education, respectively.

Of the sample, 71.6% (*n* = 302) work in a private organization, 26.8% (*n* = 113) in a public organization, 0.7% (*n* = 3) in a non-governmental organization (NGO) and finally 0.9% (*n* = 4) in another type. The tenure of the participants in their organizations was 6.95 years (*SD* = 7.86), and the mean in their jobs was 5.77 years (*SD* = 6.81). For more details on the characterization of the sample, see Table 1 in the results section. This table shows that most participants belong to economic activity, social services, health, and education. At the other extreme, we find mining and quarrying with only two people who participated in the study.

### 2.2. Instruments

The organizational dehumanization scale (ODS) proposed by Caesens et al. [39] was developed and validated in a population of 1209 workers from a variety of organizations. It consists of a single factor corresponding to the organizational dehumanization that presents 11 items (e.g., “My organization considers me as a number”) and the way to answer is in a Likert format from 1 (“strongly disagree”) to 7 (“strongly agree”). The authentication value of the instrument obtained in the original study corresponds to a Cronbach’s alpha of 0.89, and exploratory factor analysis informed factor loadings from 0.52 to 0.81, indicating that there is only one factor related to organizational dehumanization [39].

To prepare the Spanish version of the instrument, the guidelines from the International Test Commission Guidelines for Translating and Adapting Tests were followed [40]. First, an initial translation into Spanish was carried out by two experts in both languages, following the criterion of maintaining exactly the meaning of each question, varying only the necessary idiomatic turns. The reverse translation was then carried out by a native speaker with bilingual proficiency in Spanish. After verifying the equivalence of the items in both languages by experts, they were included in the general form. According to Guillemin et al. [41] and Beaton et al. [42], the whole process also implied a cultural adaptation, and adapted instruments should be used in a new population with a different country and language to ensure content validity. In line with what was previously described, the different stages of translation, synthesis, reverse translation, expert committee review, and pre-test were completed [41]. The final instrument is in Appendix A. 

### 2.3. Measures for Criterion Validity

Three measures were used to assess the criterion validity: job satisfaction, organizational citizenship behavior, and authentic leadership. Job satisfaction was included as a criterion measure because there is evidence of its negative inverse relationship with organizational dehumanization [1,39]. Indeed, it is argued that dehumanization can lead to a reduction in job satisfaction [43,44]. Job satisfaction was measured by Meliá et al. [45] Questionnaire S20/23, which consists of 23 items and is answered using a seven-point Likert response format from 7 (“very satisfied”) to 1 (“very dissatisfied”), high overall reliability was obtained (*α* = 0.82), and the component factors ranged between 0.76 and 0.89, and results that have been confirmed by subsequent studies [46,47]. 

Second, organizational citizenship behaviors were included as a criterion measure because it has been shown that dehumanization reduces the intention to provide help to others and encourages the use of violent measures in the organization (e.g., abusive supervision), which results in a negative relationship with citizenship behaviors [43,48]. Organizational citizenship behaviors were measured through the instrument developed by Rosario-Hernández et al. [49], whose theoretical basis is found in Organ’s approaches to these behaviors [50]. The scale consists of 23 items, with a Likert response format ranging from 1 (“strongly disagree”) to 6 (“strongly agree”). The total reported reliability amounts to a Cronbach’s alpha of 0.85, and the dimensions fluctuate between 0.64 and 0.82. In addition, validity is supported by factor analysis (KMO) with a value of 0.85 and the Intercorrelation between its scales and a social desirability scale.

Third, authentic leadership was included as a criterion measure since it represents the opposite pole of the leadership characteristic of dehumanization, such as petty tyranny, destructive leadership, or abusive supervision [43]. Therefore, a negative relationship between authentic leadership and organizational dehumanization is expected. On the other hand, authentic leadership exerts a positive influence on the processes of social identification of workers with their organization and workgroups [51,52,53]. Authentic leadership was assessed by the authentic leadership questionnaire (ALQ) proposed by Walumbwa et al. [54] and adapted to Spanish by Moriano et al. [55]. This instrument consists of 13 items, with a Likert response format that ranges from 0 (“never”) to 6 (“always”) to rate leaders’ attributes. The composite reliability obtained ranged from 0.84 and 0.9, which is higher than the minimum value of 0.7 that is considered adequate. In turn, satisfactory indicators of discriminant and concurrent validity were also reported [55].

Consequently, and based on what has been explained above, a negative correlation was expected between job satisfaction, organizational citizenship behaviors, authentic leadership, and organizational dehumanization.

### 2.4. Procedure 

To verify the understanding of the semantic characteristics of the instrument as well as the adequacy of the answer option, a cognitive interview was run with three workers with similar characteristics to the final sample following the guidelines of Willis [56]. In the cognitive interview conducted with three people, no problem was noted that would justify the elimination of item number one. Secondly, versions of the protocol were prepared and uploaded to the online Survey Monkey platform. Third, a list of organizations and contacts was created, and an appointment was made to obtain administrative authorization. Once these two steps were completed, data were collected.

The workers were asked for their voluntary participation in the study, which could be face-to-face or online through the Survey Monkey platform. In both instances, each participant was provided with written information about the study and an informed consent form. After reading the information and agreeing to participate by signing the form (or accepting the consent form in the digital version), the participants received a paper questionnaire and an envelope (or they answered the online form in the digital version). After completing the questionnaire, the participants delivered the sealed envelope directly to the researchers to ensure the confidentiality of their responses. In the case of the online survey, the answers came directly to the email address of the researchers. 

### 2.5. Data Analysis Strategy

First, the factor structure of the organizational dehumanization scale was contrasted using factor analysis. For this, it was necessary to divide the sample of 422 into two equitable samples (211 each) by randomly selecting the subjects to perform exploratory factor analysis and confirmatory factor analysis, respectively. The sample size was adjusted to the 200 required by Hair et al. [57] to carry out factor analysis, whether exploratory or confirmatory.

In relation to the confirmatory analysis (extraction method used: maximum likelihood, varimax rotation), the *χ*^2^/*df*, RMSEA, SRMR, CFI, and TLI indexes were used as a goodness of fit test. In this regard, values less than or equal to three for the *χ*^2^*/df* ratio are indicative of an acceptable adjustment, although it is greatly affected by the sample size [57]; RMSEA values less than 0.03 indicate an excellent fit, less than 0.05 indicate a very good fit, and less than 0.08 indicate a good fit [57]. In turn, SRMR values less than 0.08 indicate a good adjustment, although values equal to or less than 0.09 can also be accepted when there is a good adjustment in RMSEA or CFI [57]. Finally, CFI values equal to 0.95 indicate a good fit of the model [57,58], although there are also authors who maintain that values of 0.90 or even 0.80 are acceptable [59]. As for TLI, values closer to 1 indicate a better fit [60].

Regarding the convergent validity analysis, the scheme proposed by Shipp et al. [61] was followed, who suggest as indications of this validity that the item-factor should be greater than 0.70. In addition, it must be considered how many item factors are significant. Together with the previous analyses, the possible effect of the common method variance of all the items of the scales used through the Harman test was estimated [62]. Once this was done, the validity was tested, for which Pearson’s bivariate correlation analysis was used.

An analysis of the descriptive statistics of the sample was also carried out and the Cronbach’s alpha and McDonald’s omega coefficients were determined in terms of the reliability of the scales. The IBM SPSS Version 24 and IBM SPSS AMOS Version 24 programs were used for all these analyses.

## 3. Results

Table 1 shows the descriptive information of the sample. Employees from the transportation sector, storage, and communications have a higher mean age and have been working more years in the company. On the contrary, mining and quarrying had the lowest mean, both in age and time in the company, which might be caused by the physical demand of this area. 

Table 2 shows the overall descriptive statistics for all variables. Job satisfaction has the highest mean, and authentic leadership has the lowest one.

To obtain evidence about the factor structure of the instrument, the procedures of exploratory factor analysis (EFA) and confirmatory factor analysis (CFA) were applied. The sample of 422 was divided into two equitable samples by randomly selecting the answers. As seen in Table 3, the first item, “My organization makes me feel that a worker is easily as good as any other”, presents a problem in its factor loading with a value of −0.210, perhaps due to ambiguity, since it can be seen as a positive or negative quality. The EFA was then performed for 10 items, finding acceptable factor loadings for the model. In addition, it was found that the items all loaded in one factor which is consistent with the authors [39].

**Figure 1:**
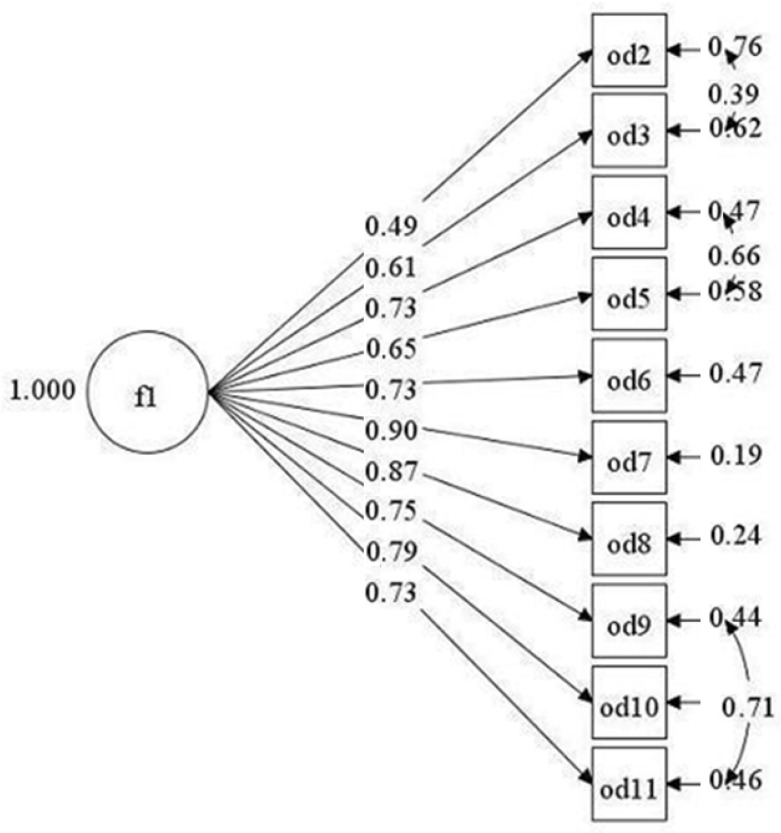
Model of the Organizational Dehumanization Scale.

Subsequently, the CFA was performed to analyze the model with a factor. Given the initial adjustment obtained, the measurement errors in items 2, 3, 4, 5, 9, and 10 had to be correlated to improve the values of the goodness indexes. This is based on the fact that items could be interpreted in a similar way as items 2–3 [63] since both refer to the perception of being replaced; the meaning of items 4 and 5 “My organization considers me as a tool to use for its own ends” and “My organization considers me as a tool devoted to its own success”, is very similar because both questions are aimed at knowing if the worker feels like a tool for the success of the organization. Finally, items 9 and 11, “My organization treats me as if I were a robot” and “My organization treats me as if I were an object”, both mention how the worker feels treated, only the last word is changed, from robot to object. This can help reduce dependency items within a scale through direct actions [64]. The corrected omega coefficient was calculated, resulting in ω = 0.90, indicating that the reliability findings are not being oversized [65]. The results are shown in Table 4, the RMSEA has a value of 0.09, CFI 0.93, TLI 0.90, and finally, the SRMR 0.05, complying with most of the criteria described above, showing a better adjustment than the original study. To conclude, in Figure 1, the estimation of the standardized parameters is given; both the factor loadings and the correlation between the factors are acceptable.

For convergent validity analysis, as seen in Figure 1, all item saturations from corresponding factors were significant (*p* < 0.01), ranging between 0.49 and 0.84, fulfilling requirements for convergent validity. Furthermore, the results of the reliability analysis (Table 5) showed that the instrument obtained internal consistency of α = 0.92 and ω = 0.92 for the organizational dehumanization scale (10 items). The result is consistent with what was delivered by Caesens et al. [39], where it shows an internal consistency of 0.94 in their case for 11 items.

Subsequently, the Harman test shows that all the items on the scales were subjected to an exploratory factor analysis with the principal component’s method and varimax rotation, forcing extraction to a single factor. Thus, it was evaluated whether the participants were able to distinguish between the different scales of the study. The result was favorable, obtaining an explained variance of 30.52, which is less than 50% of the common variance, so the effect of the common variance does not seem to significantly affect the relationships between the variables studied.

To test the criterion validity for the Spanish version of the ODS, correlations analyses were conducted among the organizational dehumanization, authentic leadership, job satisfaction, and organizational citizenship behaviors. According to Table 5, job satisfaction, organizational citizenship behaviors, and authentic leadership were negatively related to the perception of organizational dehumanization. In total, these findings indicate that the ODS has acceptable criterion-related validity.

## 4. Discussion

The objective of this study was to translate, adapt, and validate the Spanish version of the organizational dehumanization questionnaire by Caesens et al. [39]. The psychometric properties have been analyzed, and the results confirm the factor structure of Caesens et al. [39]. Exploratory and confirmatory factors confirm that the questionnaire has a one-factor structure. Item number 1 was deleted: “My organization makes me feel that a worker is easily as good as any other”, due to problems in the factor loading and because of the differences in interpretation between English- and Spanish-speaking subjects. In the English scale, this item was probably interpreted as something positive, while in the Spanish validation, this item was questioned by the panel of expert judges due to its ambiguous meaning and finally by the subjects (as it could be considered as a positive or negative aspect). Reliability and internal factor validity were good.

Another psychometric aspect especially addressed in this study was content validity. Following the guidelines of the International Test Commission Guidelines for Translating and Adapting Tests [40] and in attention to the importance of a relevant translation process of questionnaires for cross-national surveys [66], stages of translation, synthesis, reverse translation, expert committee review, and pre-test were completed [41]. Thus, the sociocultural adaptation of the items, a critical factor in the entire translation process, was achieved. 

Concurrent validity was demonstrated. As expected, the ODS showed significant and negative correlations with job satisfaction, authentic leadership, and organizational citizenship behaviors. These findings are consistent with previous research that confirms the negative association with job satisfaction [39]. Additionally, the negative relationship between authentic leadership and organizational citizen behaviors is theoretically consistent. This study is an empirical contribution since, as far as we know, there is no empirical evidence available about the relationship between these variables and organizational dehumanization. 

The present research releases the ODS by Caesens et al. [39] in Spanish and shows that job satisfaction, authentic leadership, and organizational citizenship behaviors have a negative relationship with organizational dehumanization. To the best of our knowledge, this study is the first validation in the Spanish language and, therefore, contributes to the organizational psychology literature. Organizational dehumanization is understood as the experience that employees have when they are objectified, personal subjectivity is denied, and they are perceived as a “tool” or instrument for the interests of the organization. The most common form of dehumanization within an organization refers to the intentional mistreatment of employees, abuse, and involuntary contempt against their well-being [1]. Dehumanization has been described as a frequent experience in employees in modern organizational contexts [14] but more research is needed on this phenomenon and its consequences at individual and organizational levels. We have the knowledge that organizational dehumanization is related to the configuration of work design [67], with the individual redesign processes such as job crafting [68] or different styles of leadership beyond leadership [69]. Furthermore, it has been shown that perceived organizational support negatively predicts organizational dehumanization [39] and that organizational dehumanization mediates the positive relationship between perceived organizational support and well-being. Furthermore, Arriagada-Venegas et al. [70] showed the moderator role of organizational dehumanization between authentic leadership and organizational citizenship behavior, and Arriagada-Venegas et al. [71] confirmed the mediator role of organizational dehumanization between authentic leadership and job satisfaction. Additionally, Arriagada-Venegas et al. [72] demonstrated that the ingroup–outgroup relationship influences the perception of organizational dehumanization and that gender and status have moderating roles, being low-status females at most risk of perceiving themselves as dehumanized. Following the results of recent studies that confirmed the influence of the leader in OD, and the relationship between OD in the employee’s perception, well-being, psychological strains, absenteeism, turnover intentions, and affective commitment within other negative consequences [73,74,75,76,77], future research should consider other relevant variables that are influenced by organizational dehumanization, such as innovation capability [78], self-efficacy and workplace well-being of the employees [79], service flexibility and service climate [80], and cognitive dissonance [81,82].

## 5. Conclusions

First, this study provides a validated assessment tool for organizational dehumanization in the Spanish-speaking population that allows further understanding of a phenomenon of increasing scientific interest, which, as indicated, starts from the theories of the sociologists Marx [3], Durkheim [4], and Weber [5], going through social psychology with the theories of infrahumanization [6,7] and the dual model of dehumanization [8] which is finally introduced in organizational environments to understand how organizations affect the perception of workers in feeling like an object. Second, having a reliable instrument allows organizations to obtain feedback on organizational dehumanization perceived by employees. As we stated previously, dehumanization has a negative impact since it reduces the tendency of managers to adopt others’ point of view, increases interpersonal distance and the use of harmful behavior toward the employees, and justifies the use of these violent and harmful means [21]. Dehumanization increases avoidance behaviors and social rejection and reduces the intention to provide help to the employees [26,27]. It is important for organizations to be aware of this phenomenon when implementing human resources practices, organizational programs, and policies. Third, this study demonstrates the importance of organizational dehumanization over other processes. The results show the negative consequences of dehumanization despite having leaders who are authentic and how job satisfaction and citizenship behaviors can be overshadowed by the presence of organizational dehumanization.

With respect to companies, avoiding organizational dehumanization at work has plenty of positive and significant outcomes. As it was stated before, dehumanization increases the level of anxiety or depression of the employees. Dehumanization decreases the necessity for competition and interaction with other employees and damages the well-being of the employees who suffer [14]. Furthermore, dehumanization promotes the perception of burnout, negatively affects the worker’s citizenship behaviors, and leads to emotions of sadness, anger, guilt, and shame [14,30]. Taking this into consideration, measuring organizational dehumanization will have practical repercussions since companies with low levels of dehumanization will decrease the number of employee absences, rotation, and mental health diseases.

In terms of the applied implication of the study results, at least two lines of potentially useful practical proposals can be pointed out for the companies. First, organizations should focus on how to improve the perception of humanization of the employees as this is affecting job satisfaction and citizenship behaviors. Furthermore, the leadership style promotes organizational humanization. For example, the current study shows the negative relationship between organizational humanization and authentic leadership, and Arriagada-Venegas et al. [71] showed that authentic leadership influences organizational dehumanization and therefore affects citizenship behaviors. Companies can focus on designing more humane organizations, implementing corrective actions to analyze whether there is organizational dehumanization, and, if so, reducing the perception of dehumanization (e.g., changing the style of leadership to authentic leadership). Second, practical proposals are suggested at the level of people management in the organization that promotes this humanized treatment. Specifically, in the selection of future managers or by viewing employees as unique people instead of a number or machine for the purposes of the organization as this may impact the results of the company (e.g., increased extra-role behaviors of support and organizational citizenship behaviors, greater job satisfaction, higher retention of talent, etc.).

This study has shown important results as it provides a validated assessment tool to assess the perception of organizational dehumanization in the Spanish-speaking population. The scale has been shown to have good psychometric properties, and it is concluded that ODS can be used in Spanish-speaking countries (e.g., Latin America and Spain) and is a useful instrument to identify high levels of organizational dehumanization in the work context, functioning as the first step in the development of appropriate prevention or intervention strategies. 

## 6. Limitations

The study has certain limitations. First, the answers to the questionnaire could be influenced by the need for social desirability of the employees. Secondly, a snowball sampling technique was adopted to recruit participants, and most of them came from the same region, which limits the generalization of the results. Third, a more heterogeneous sample selection in terms of geographic characteristics is required to determine whether the results are generalized to a larger sample in the Spanish-speaking community. Fourth, in this study, the scale behaves in the same way in both Spanish and English, except in the case of item 1, where the item was understood ambiguously in the Spanish version. It shows that the cultural aspects that could be considered influential in determining the responses of the participants have not been confirmed. This is the first approximation that we make of the scale in a Spanish-speaking cultural context, and we think it would be appropriate for the scale to be applied in other Spanish-speaking contexts to verify the validity of our results. Fifth, with the local nature of the sample, one should be cautious about generalizing the findings to all Chilean workers. Moreover, this study and these tests were not designed to test causal relationships; it was meant to study potential relationships through correlations. These limitations should be considered in the future application of the questionnaire. 

## Figures and Tables

**Table 1:** Descriptive statistics according to economic activity.

Area	Age			Time in the Organization (Years)		Gender (%Man)
	*n*	*M*	*SD*	*M*	*SD*	
Agriculture, livestock, hunting, fishing, and forestry	5	31.40	4.93	6.83	6.55	60.0%
Mining and quarrying	2	30.00	7.07	3.17	3.06	100.0%
Manufacturing industry	73	37.95	11.18	6.55	6.93	69.9%
Electricity, water, or gas supply	11	40.18	13.46	6.36	9.35	72.7%
Building	20	39.00	13.60	4.75	5.26	75.0%
Commerce, hotels, and restaurants	75	35.59	11.10	4.30	5.48	34.7%
Transportation, storage, and communications	59	43.59	11.83	9.93	10.16	52.5%
Financial services	24	35.71	13.34	5.65	8.20	66.7%
Public administration and defense	12	39.33	10.05	7.32	6.35	66.7%
Social services, health, and education	106	40.12	10.30	7.84	7.82	30.2%
Other services	35	40.29	10.18	8.15	9.42	62.9%
Total	422	38.96	11.40	6.95	7.86	50.7%

Note: *n* = 422; *M* = mean; *SD* = standard deviation.

**Table 2:** Descriptive statistics of the study variables.

	Minimum	Maximum	*M*	*SD*
Authentic leadership	0	6	4.21	1.49
Organizational dehumanization	1	7	4.41	1.39
Job satisfaction	1.13	7	5.21	1.18
Organizational citizenship behaviors	2.43	6	4.76	0.56

Note: *n* = 422; *M* = mean; *SD* = standard deviation.

**Table 3:** EFA factor loadings for 11 and 10 items.

Item	Factor Loadings	Factor Loadings
OD 1	−0.21 *	-
OD 2	0.49 *	0.48 *
OD 3	0.57 *	0.56 *
OD 4	0.70 *	0.71 *
OD 5	0.69 *	0.70 *
OD 6	0.80 *	0.81 *
OD 7	0.90 *	0.90 *
OD 8	0.89 *	0.90 *
OD 9	0.74 *	0.70 *
OD 10	0.80 *	0.77 *
OD 11	0.74 *	0.69 *

Note: *n* = 211. * *p* < 0.01.

**Table 4:** Results of the CFA of the Organizational Dehumanization Scale.

	*χ* ^2^	df	*χ*^2^/df Ratio	SRMR	RMSEA	CFI	TLI
Organizational dehumanization	82.11	32	2.57	0.05	0.09	0.93	0.90

Note: *n* = 211. SRMR = standardized root-mean-square residual; RMSEA = root-mean-square error of approximation; CFI = comparative fit index; TLI = Tucker–Lewis index.

**Table 5:** Matrix of correlations between the study variables and reliability indexes.

Variable	1	2	3	4
1. Authentic leadership	(*α* 0.97) (*ω* 0.98)	−0.28 **	0.61 **	0.25 **
2. Organizational dehumanization		(*α* 0.92) (*ω* 0.92)	−0.30 **	−0.12 *
3. Job satisfaction			(*α* 0.96) (*ω* 0.96)	0.33 **
4. Organizational citizenship behaviors.				(*α* 0.84) (*ω* 0.87)

Note: *n* = 422. Cronbach’s alpha and McDonald’s omega are presented diagonally * *p* < 0.05. ** *p* < 0.01.

## Data Availability

The data presented in this study are available on request from the corresponding author. The data are not publicly available due to privacy and are available from the corresponding author upon reasonable request.

## Appendix A

The validated scale.

Spanish Version of the Organizational Dehumanization Scale.

Instrucciones: por favor, indique con un O o con una X el número en la escala de 1 a 7 que mejor refleja su grado de acuerdo con cada una de las siguientes afirmaciones. Tenga en cuenta que 1 significa totalmente en desacuerdo y 7 totalmente de acuerdo.

**Table A1:** Versión en español de la Escala de Deshumanización Organizacional.

	Enunciados	Total Desacuerdo	Grado							Acuerdo total
1.	(Removed) Mi organización me hace sentir que un trabajador es tan bueno como cualquier otro.	Total desacuerdo	1	2	3	4	5	6	7	Acuerdo total
2.	Mi organización no dudaría en reemplazarme si eso permitiera a la compañía obtener mayores beneficios.	Total desacuerdo	1	2	3	4	5	6	7	Acuerdo total
3.	Si mi trabajo pudiera ser realizado por una máquina o un robot, mi organización no dudaría en reemplazarme por esta nueva tecnología.	Total desacuerdo	1	2	3	4	5	6	7	Acuerdo total
4.	Mi organización me considera como una herramienta a usar para sus propios fines.	Total desacuerdo	1	2	3	4	5	6	7	Acuerdo total
5.	Mi organización me considera como una herramienta para su propio éxito.	Total desacuerdo	1	2	3	4	5	6	7	Acuerdo total
6.	Mi organización me hace sentir que lo único importante es mi desempeño laboral.	Total desacuerdo	1	2	3	4	5	6	7	Acuerdo total
7.	Mi organización sólo está interesada en mí cuando me necesita.	Total desacuerdo	1	2	3	4	5	6	7	Acuerdo total
8.	Lo único que cuenta en mi organización es lo que yo pueda contribuir a esta.	Total desacuerdo	1	2	3	4	5	6	7	Acuerdo total
9.	Mi organización me trata como si fuera un robot.	Total desacuerdo	1	2	3	4	5	6	7	Acuerdo total
10.	Mi organización me considera como un número.	Total desacuerdo	1	2	3	4	5	6	7	Acuerdo total
11.	Mi organización me trata como si fuera un objeto.	Total desacuerdo	1	2	3	4	5	6	7	Acuerdo total
